# Management of lumbar bone stress injury in cricket fast bowlers and other athletes

**DOI:** 10.17159/2078-516X/2023/v35i1a15172

**Published:** 2023-06-05

**Authors:** JW Orchard, R Saw, A Kountouris, D Redrup, P Farhart, K Sims

**Affiliations:** 1 School of Public Health, University of Sydney, Australia; 2 Australian Institute of Sport, ACT, Australia; 3 LASEM Research Centre, LaTrobe University, Australia; 4 Cricket NSW Homebush New South Wales, Australia; 5 School of Sport, Exercise and Rehabilitation, University of Technology, Sydney, New South Wales, Australia; 6 School of Health and Rehabilitation Sciences, University of Queensland, Australia

**Keywords:** Lumbar spine, stress fracture, spondylolysis, spondylolisthesis, cricket, fast bowling, bone marrow oedema

## Abstract

**Background:**

Recent guidelines (including a special series in *The Lancet*) have emphasised a minimal role for imaging when assessing low back pain in adults, as the majority of patients will have non-specific findings on imaging that do not correlate well with pain.

**Objective:**

To assess whether the diagnosis of lumbar bone stress injuries in young athletes should be considered an exception to the recommendation to avoid imaging for low back pain in adults.

**Method:**

Narrative review.

**Results:**

Early lumbar bone stress injury diagnosis has been available via traditional MRI sequences (and its precursor Single Photon Emission Computed Tomography (SPECT)) for 25–30 years. MRI assessments using bone window sequences (such as Volumetric Interpolated Breath-hold Examination (VIBE)) have allowed a better understanding of the diagnosis and prognosis of lumbar bone stress injury in young athletes. MRI with bone sequences has allowed non-radiating scans to serially follow the healing of unilateral stress fractures. In the majority of cases, non-chronic unilateral fractures can heal; however, this takes three-six months rather than the six-ten weeks that would be the typical unloading period if using symptoms (only) as a guide. The use of MRI to provide evidence of bony healing (as opposed to fibrous union, which creates the pars defect that predisposes to further bone stress lesions) can lead to better long-term outcomes in athletes. There is evidence to flag this as a structural lesion which is both painful and, more importantly, can heal/resolve if managed correctly. Therefore it represents an important ‘specific’ diagnostic subset within adult low back pain.

**Conclusion:**

Structural (rather than functional) management of bone stress injuries in high-demand athletes, such as cricket pace bowlers, is in contrast to the recommendation of functional management for general back pain in adults. Structural management is justified when there are demonstrable superior outcomes of having better structure. Although this has not yet been shown in randomised trials of elite athletes, apparent lengthier Test cricket careers of pace bowlers who do not have pars defects suggest better athletic outcomes if bony healing is achieved. For lower demand young adults, or athletes with established bilateral pars defects, functional management may be more pragmatic.

Lumbar bone stress injuries (LBSI) have been recognised as a common cause of back pain in cricket fast bowlers and other athletes for at least 40 years.^[1]^ Spondylolysis (including active LBSI and chronic pars defects) has been reported to occur in up to 44% of professional athletes, which is significantly more common than in amateur athletes and non-athletes. ^[2]^ Spondylolysis has consistently been found to be the most common cause of back pain in young athletes.^[3]^

The recognition and management of LBSI has evolved with improvements in research and knowledge, plus in part, with improvements in medical imaging. ^[4]^ Over the time period from the 1980s to the present, there have been substantive advances in imaging (see Table 1). When X-ray was the only available imaging modality, lumbar stress fractures (spondylolysis) were only visible when they had already occurred and then failed to heal. Further to this, if they become bilateral they might lead to spondylolisthesis (slippage), also visible on X-ray. Spondylolisthesis was graded 1–3 on X-rays and Grade 3 slips were relatively common in athletes in years prior to 2000.^[5]^

Whilst the introduction of computerised tomography (CT) scanning allowed easier identification of the cortical breach LBSI of the pedicle and pars interarticularis, it is important to note that CT possesses reduced sensitivity in diagnosing non-cortical breach LBSI (stress reactions).^[6]^ The introduction of nuclear imaging, known as Single Photon Emission Computed Tomography (SPECT) allowed stress reactions and pre-fracture pathology to be identified as ‘hot spots' in the 1990s and 2000s. More recently, MRI assessment using bone window sequences (such as Volumetric Interpolated Breath-hold Examination (VIBE)) has become the preferred form of imaging.^[7]^

## Back pain in middle-aged adults

It is important to consider that lumbar stress fractures are one of many causes of back pain, with an important distinction that lumbar stress fractures almost always affect athletic teenagers and young adults, whereas back pain itself is a common symptom at all ages. Despite the advances in lumbar imaging, there is widespread evidence to show that this has not led to an overall improvement in the management of back pain in adults.^[8, 9]^ The importance of imaging (of back pain in most adults) is to rule out ‘sinister’ causes, such as tumours or conditions leading to significant neurological compromise. These conditions are uncommon and are visualised in less than 5% of lumbar scans (including 3D imaging, such as CT and MRI). In the vast majority of middle-aged or older adults, substantial lumbar MRI changes will be present in most of the population and represent ‘normal age-related (degenerative) changes’. They do not correlate well with back pain, being present in asymptomatic people (of the same age) and generally as often as in symptomatic patients. The presence of old fibrous unions (pars defects) from unhealed stress fractures in youth are an example of imaging findings in middle-aged adults that may not correlate with symptoms. For this reason, unless there are ‘red flag’ symptoms which alert to the possibility of sinister pathology, most guidelines recommend avoiding imaging in uncomplicated middle-aged back pain. The concern in middle-aged patients is that exercise is usually the most evidence-based treatment modality for back pain, but there is the tendency for patients to avoid loading if they have been told they have degenerative changes on scan, such as disc degeneration and prolapses. When medical imaging is performed, the practitioner should explain what findings are relevant, and which findings are likely to be ‘normal’ for the person’s demographic.

## Natural history of stress fractures in the X-ray era

Prior to the 2000s, most fast bowlers in their late teenage years sustained a lumbar stress fracture, or more than one, and generally, they did not take much time off. The vast majority ended up with chronic fibrous unions. Spondylolysis was so common that in the early 1980s, a cohort of senior Victorian squad members was assessed with X-rays, which found 11 out of 12 pace bowlers had X-ray evidence of unilateral or bilateral pars defect +/− spondylolisthesis.^[10]^ In the early 1990s, a cohort of junior Western Australian fast bowlers (mean age of 17) exhibited a prevalence of 55% for the presence of a spondylolisthesis or pars defect.^[11]^ Some of the bowlers from this era coped well, others had chronic niggling back pain for their entire career, with a reduced ability to bowl at extreme pace, and a small percentage needed to retire. Spondylolysis and spondylolisthesis are also very common amongst athletes in other sports.^[9, 12]^

## Specialist management of stress fractures in the CT/bone scan era

The emergence of the CT scan (3D imaging) and bone scan (functional assessment of bone stress), combined as SPECT-CT, allowed a much earlier diagnosis of lumbar stress fractures from the 1990s onwards. The net result of this was that high grade (2 or 3) spondylolisthesis in athletes became a less common occurrence, as athletes were encouraged to rest upon a diagnosis of an acute painful stress fracture. Generally, rest/unloading was prescribed until symptoms resolved, which was usually a period of 6–10 weeks. Although high grade spondylolisthesis becomes less common, spondylolysis (pars defects) did not appear to be eliminated by the routine use of the CT scan.^[13]^ Because there is a high amount of radiation associated with SPECT-CT, it was difficult to justify follow-up scans to monitor for a bony union. Lumbar bone stress injuries and consequent non-unions remained high in this era.^[14]^

## Management options of bone stress injury in the MRI era

Treatment options in the MRI era have expanded now that tools are available to assess the presence, extent and intensity of bone marrow oedema within the posterior elements of lumbar vertebrae.^[15]^ MRI sequencing techniques have developed further in recent years with the advent of special bony windows (thin slice three-dimensional T1-weighted radiofrequency spoiled echo sequences, including volumetric interpolated breath-hold examination (VIBE) sequence) and have allowed ‘CT-like’ bony imaging with MRI.^[7]^ One study found MRI with VIBE sequences to be 98% sensitive and 92% specific for the diagnosis of LBSI compared to CT.^[6]^

Poor quality MRI images without the correct sequencing protocols can lead to misdiagnosis and mismanagement from the outset. Radiologists and MRI technicians with considerable experience in the imaging of LBSI are preferred. An MRI protocol should include a heavily water weighted sequence, such as a short-tau inversion recovery (STIR) sequence, to detect bone oedema, along with a fine-slice VIBE or equivalent sequence to assess for a fracture.^[7]^ The major advance has been the ability to follow serial MRI scans to monitor for the resolution of bone oedema and bony healing given that radiation is not a concern.^[16]^

Resolution of bone oedema correlates well with bone healing on CT, and resolution of clinical symptoms. ^[17]^ Another study found MRI with VIBE sequencing can be used to monitor healing in cricketers, and that recurrent fractures take longer to radiologically unite. ^[18]^

## Bone stress injury without fracture

Bone stress injury, as demonstrated on MRI, without any cortical breach (as seen on a bone sequence view of an MRI or CT scan) is a condition to be treated with caution. It is known that this is a precursor lesion to an actual fracture, so that an athlete who continues to load without a fracture is at high-risk of progression to a fracture.^[16, 19]^ Where there is a desire from the athlete to return to ‘high-risk’ activities such as cricket pace bowling, a follow-up MRI after a period of unloading can hopefully demonstrate the return of signal ratios to normal levels, after which time it may be considered safe to resume training. ^[15]^

## Early diagnosis of unilateral acute stress fractures

When managing LBSI in Australian pace bowlers, our current approach in most situations is to not allow a return to bowling until the MRI scan (with VIBE bony window sequence) shows complete bony healing.^[4]^ Most of the time this can be achieved if the fracture (usually on the contra-lateral side to the bowling arm side) is picked up early enough ^[18]^ However, we generally find that it takes 4–6 months to get complete resolution.^[4]^ An argument in favour of this approach has been our current stock of Test bowlers, who have generally been managed this way (early MRI imaging and then unload until structural healing occurs) on multiple occasions to achieve bony union. Australian Test bowlers are required to, and generally can, bowl through high workloads at high pace. We are aware that the cohort level of evidence is not as strong as randomised trial evidence, but also that other experts take a similar approach to management internationally.^[20]^

While obtaining a high-quality MRI scan at the point of initial unilateral partial stress fracture has facilitated a process to allow many fast bowlers to achieve bony union, it is certainly not a miracle cure. Getting bony union requires many months off bowling (usually a full season) and results in some secondary temporary loss of bone density,^[21]^ meaning that recurrent stress fractures the following year are also common. Recurrent stress fractures also appear to take longer to achieve bony healing.^[18]^ Therefore, in the event of finding a stress fracture very early, it is sometimes a difficult decision for the athlete to take a long period of time off sport in the hope of better results down the track. This decision is easier to justify in the aspiring Test bowler, but can become more difficult in T20-focused bowlers, amateur players, non-bowlers and in other sports with lower demands, where the long-term benefits of superior bony union may be less relative to the downside of a long layoff period. Ages from 23.1 to 24.9 years have been reported as the 95% confidence interval for the attainment of peak bone mineral density in males ^[22]^, and if you can make it to this age without any established spondylolysis or spondylolisthesis, it seems to benefit the second half of an athletic career. In particular, we have not seen as many cases of bowlers requiring premature retirement due to chronic back pain in the modern era compared to the 1980s, 1990s and 2000s.

In the post-athletic career, almost the entire population will have significant degenerative changes in the lumbar spine by middle age.^[23]^ There is no clear evidence to say that those who suffered lumbar stress fractures in youth have any more back pain in middle-age than those who didn’t. Some studies have shown equivalent or better pain in middle-age between retired athletes and the general population, including athletes with spondylolysis and spondylolisthesis.^[9, 24]^

## Bilateral stress fractures

When there is an established non-union in the pedicle or pars interarticularis contralateral to the bowling arm side, a stress fracture to the ipsilateral side, that is the bowling arm side, appears more likely to occur, often in the pedicle. Bony healing remains desirable, but even with prolonged rest and optimal management, this outcome may not occur in the presence of an established non-union on the other side.^[25–27]^

A chronic lesion that is very unlikely to heal can be managed more pragmatically with a return to activity as pain allows. Treatment is generally then ‘functional’ (taking a shorter period of time off in line with pain flare-ups) rather than structural, with the concession that there may be limits on workload tolerance or pace in the longer term. Very occasionally, surgery can be indicated with bilateral lesions leading to chronic pain which prevent bowling at the desired level.^[28]^ Because of the high morbidity associated with surgery, this is usually only an option when retirement is being considered (i.e. that surgery should not be considered routine but is instead a career-saving procedure).

## Use of bracing

Thoracolumbar spine bracing has often been included as part of the traditional specialist management protocol of spondylolysis,^[29]^ although recommendations for bracing are not universal.^[30]^ While the application of a brace to limit lumbar extension and rotation logically should promote healing, there is a lack of strong evidence to support a clear advantage in all athletes. A meta-analysis of the conservative management of spondylolysis with Grade 1 spondylolisthesis did not find a difference on return to sport or clinical outcomes for those treated with or without a brace.^[31]^ There may be benefit for specific individuals from encouraging or enforcing unloading from sport. There may be a subset of athletes that are more likely to benefit from bracing, including those with persisting pain at rest, exaggerated lumbar lordosis, clinical factors making delayed or non-union more likely, or bilateral stress fractures.^[31]^ In practice, we generally do not prescribe braces for full-time athletes. The “real-world” advantage of bracing may be in the schoolkid who might still play hours of casual twisting sport at school (without a brace) even despite agreeing to refrain from formal sporting competition. With an initial diagnostic management protocol and enforced unloading +/− bracing, good functional results can be achieved in a majority of cases, but it is also to be expected that spondylolysis defects will persist in a high percentage of athletes.^[29]^

## Indication for MRI scan

**Fig. 1:**
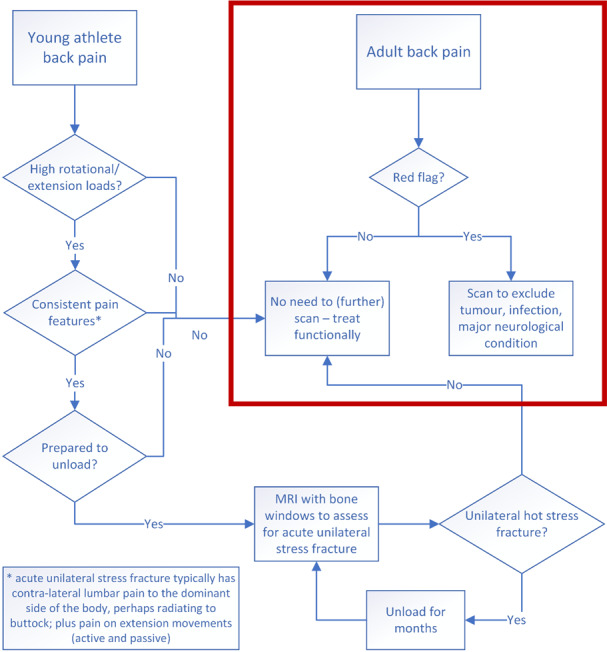
Flowchart summary. The bordered section represents scan recommendations from adult guidelines for low back pain.^[8] ^We propose that a sub-section of adults (young athletes) needs further consideration of scan requirements, as structural management in this subgroup can lead to improved results. A unilateral “hot” stress fracture is one with bone marrow oedema around an acute fracture line, which has the potential to heal if unloaded.^[18]^

Figure 1 and Table 2 can assist with the diagnostic approach for back pain in the young athlete. The most difficult question to start with is which athletes with back pain warrant an MRI scan. Our view is that an MRI scan should be used when the yield is high for finding the lesion which may be amenable to healing, in the athlete who would be prepared to undertake the necessary unloading to allow healing. In the sport of cricket, young pace bowlers with contra-lateral side back pain associated with bowling generally represent a high yield population for which an early scan provides value.^[32]^ In the same sport, specialist batters who do not bowl (or play any other twisting sport) are far less likely to have bone stress lesions and hence do not usually warrant early referrals for an MRI scan. Different sports will have varying yields for early scans. A recent review in the sport of baseball found that laterality in pain that lasted for over 4 weeks, which interfered with running and with spinous process tenderness were the characteristics most associated with spondylolysis.^[33]^

## Conclusion

Management of lumbar bone stress injury is complicated as there are no clear pathways that can be directed by Level 1 evidence. Randomised control trials in elite athletes are very difficult to conduct, and we believe that elite athlete management needs to be different for the general community (e.g. Table 2, Figure 1). The trend towards managing back pain with a functional approach makes sense in low-demand young athletes, but we strongly believe that it risks career-shortening in high-level young athletes. In this group, which includes cricket fast bowlers, we advocate a structural approach.^[4]^

## Figures and Tables

**Table 1:** Imaging options in athletic back pain over the past 50 years

Imaging modality	Advantages/disadvantagesof imaging	Time frame/ availability
Plain X-ray with oblique views	Able to see chronic changes (only) including pars defects and spondylolisthesis. Cheap.	Available from 2^nd^ half of 20^th^ Century.
CT scan with SPECT	Able to visualise acute bone stress (nuclear medicine component) and 3D structural bone defects (CT component). Relatively high levels of radiation involved.	Available from 1990s
MRI scan with appropriate sequencing (STIR or equivalent to identify bone oedema, andVIBE or equivalent to assess for fracture line)	Able to visualise acute bone stress and 3D structural bone defects (from bone sequences). Non-radiating so can be safely repeated	Bone sequences have been available from 2010s

SPECT, Single Photon Emission Computed Tomography; MRI, Magnetic Resonance Imaging; STIR, short-tau inversion recovery; VIBE, Volumetric Interpolated Breath-hold Examination.

**Table 2:** Recommended management protocols for lumbar bone stress lesion in the young athlete

Management protocol	Imaging requirements	Management	Consequences	Recommended utility
Functional [8]	Nil	Refrain from sport when in pain, but graded return to play as soon as pain settles	Many lumbar stress fractures will remain unconfirmed; risk of spondylolisthesis if heavy loading persists when fracture is not healed	This protocol reflects guidelines for back pain in middle-aged people; appropriate in young adults who have low sporting demands
Traditional conservative treatment (Pain management) ^[29]^	SPECT-CT or MRI for diagnosis	6–10 week (approx.) of dedicated unloading +/− bracing after diagnosis of acute stress fracture	Lumbar stress fracture diagnoses will be made. Healing is not generally monitored so recurrence, pars defects and spondylolisthesis maybe more likely.	May be appropriate where the athlete prefers a faster return to sport, and acknowledges the higher risk of long term consequences. Less recommended for ‘high risk’ athletes
Bony-healing-dependent conservative treatment (structural management)^[4, 20]^	MRI for diagnosis and serially to demonstrate bony healing	Unloading from all high-risk activities until full bony healing is demonstrated (usually 4–6 months) or the nature/progress shows that bony healing will not occur	Bony healing can be achieved with early (unilateral lesion) diagnosis	Recommended in the majority of high-level athletes. In particular those at greater risk of complications (recurrence, pars defects, spondylolisthesis) which could negatively impact on their athletic career

SPECT, Single Photon Emission Computed Tomography; MRI, Magnetic Resonance Imaging
